# Urbanization is associated with non‐coding polymorphisms in candidate behavioural genes in the Eurasian coot

**DOI:** 10.1002/ece3.10572

**Published:** 2023-10-01

**Authors:** Amelia Chyb, Radosław Włodarczyk, Joanna Drzewińska‐Chańko, Jan Jedlikowski, Kimberly K. O. Walden, Piotr Minias

**Affiliations:** ^1^ Department of Biodiversity Studies and Bioeducation, Faculty of Biology and Environmental Protection University of Łódź Łódź Poland; ^2^ Faculty of Biology, Biological and Chemical Research Centre University of Warsaw Warsaw Poland; ^3^ Roy J. Carver Biotechnology Center University of Illinois at Urbana‐Champaign Urbana Illinois USA

**Keywords:** candidate genes, circadian rhythm, CKIɛ, cognition, CREB1, Eurasian coot, urbanization

## Abstract

Extensive transformation of natural land cover into urbanized areas enhances accumulation of phenotypic differences between animals from urban and nonurban populations, but there is little information on whether these changes, especially in terms of animal behaviour and circadian rhythm, have a genetic basis. The aim of this study was to investigate genetic background of behavioural differences between four pairs of urban and nonurban populations of a common waterbird, the Eurasian coot *Fulica atra*. For this purpose, we quantified polymorphisms in personality‐related candidate genes, previously reported to be associated with avian circadian rhythms and behavioural traits that may be crucial for urban life. We found general associations between landscape urbanization level and polymorphisms in 3′UTR region of CREB1 gene encoding transcriptional factor, which participates in development of cognitive functions and regulation of circadian rhythm. We also found significant differentiation between urban and nonurban populations in the intronic region of CKIɛ gene responsible for regulation of circadian clock. Although we lacked evidence for linkage of this intronic variation with coding polymorphisms, genetic differentiation between urban populations was significantly stronger at CKIɛ intron compared with neutral microsatellite markers, suggesting possible local adaptations of CKIɛ expression regulation to specific urban sites. Our results indicate that behavioural differentiation between urban and nonurban coot populations may be the effect of habitat‐specific selective pressure resulting in genetic adaptations to urban environment and supporting the microevolutionary scenario. These adaptations, however, prevailed in non‐coding regulatory rather than coding gene regions and showed either general or local patterns, revealing high complexity of associations between behaviour and landscape urbanization in birds.

## Graphical abstract

In the study, we identified polymorphisms in behavioural genes in four pairs of urban and nonurban populations of the Eurasian coot (a common waterbird) to test for their associations with landscape urbanization level. For the study, we have chosen 10 candidate genes previously reported to be associated with aggression, exploratory behaviour, circadian rhythm, cognition and migration in birds. We showed conspicuous associations between CREB1 3′UTR genotypes and the level of landscape urbanization and also recorded strong differentiation at CKIɛ intronic region among urban populations, either suggesting local adaptations through linkage with polymorphisms in coding regions or adaptations of gene expression regulation to particular urban sites.

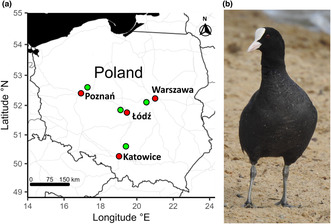

## 1 INTRODUCTION

Constant expansion of urbanized areas, increasing human population density, development of industry, transformation of natural habitats into agricultural land and intensifying word‐wide touristic movement cause a progressive shrinkage of natural living space for animals at the global scale (Seress & Liker, 2015). Most animal species are not able to effectively cope with multiple stressors generated by human‐dominated environments and become urban avoiders (Palacio, 2020). Therefore, urbanization processes are usually associated with reductions in species diversity, declines in local community richness and negative changes in animal population dynamics (Devictor et al., 2007). However, highly plastic species may adapt to increasing human pressure and effectively exploit urban resources, such as anthropogenic food supplies (Callaghan et al., 2019). This variation in adaptive abilities is also apparent at the intraspecific level, resulting in the differentiation of physiological, morphological, behavioural and reproductive traits between urban and nonurban populations (e.g. Evans et al., 2009, 2010).

Behavioural responses and stress tolerance are considered key determinants of successful colonization and persistence in urbanized areas (Caizergues et al., 2022). Birds from urban populations often show higher level of aggression towards both humans and conspecifics, increased boldness, greater risk‐taking behaviour and reduced anxiety, which is manifested, among the others, by shorter flight initiation distance (Evans et al., 2010). Other characteristics of birds adapted to urban habitats include more exploratory behaviour (Caizergues et al., 2022) and better problem‐solving skills (Audet et al., 2016). Many studies show that individuals from urban populations are faster in exploration of food sources, better adapt to novel foraging techniques and show higher efficiency in resource use (Robertson et al., 2010; Sol et al., 2011). Urban individuals may also exhibit increased sedentariness (Kark et al., 2007), probably as a result of milder microclimatic conditions in city centres during winter (so called heat islands), which may cause serious disruptions in annual cycles (Bonnet‐Lebrun et al., 2020). Finally, high level of artificial light at night (ALAN) prevails in urbanized landscape, affecting bird activity patterns and promoting alterations in circadian and circannual rhythmicity, such as earlier onset of dawn song (Miller, 2006).

Phenotypic plasticity and genetic adaptations are recognized as two major mechanisms which may generate behavioural adjustments to urban environment (Gill & Brumm, 2014). Environmentally induced changes in gene expression may enhance much faster adaptation to urban landscape (e. g. by DNA methylation) than heritable changes in DNA sequence (Watson et al., 2021). Therefore, phenotypic plasticity (mediated by gene expression) is predicted to play an important role at the early stages of urbanization processes and facilitate phenotypic adaptations, which may then be fixed at the genetic level via microevolutionary mechanisms, that is through the processes of natural selection (Yeh & Price, 2004). Finally, some individuals may be genetically pre‐adapted to urban environment (so‐called genotype sorting; Partecke, 2014) and become precursors of urban colonization (consistent with the founder effect; Clegg et al., 2002). Under this scenario, genetic differentiation between urban colonizers and source nonurban population may become apparent from the very moment of an urban colonization event (Partecke, 2014). Irrespectively of the initial level of population differentiation, two non‐exclusive evolutionary processes may shape genetic structure of urban and nonurban meta‐populations: (i) genetic drift, which randomly affects both adaptive and selectively neutral genetic markers; (ii) natural selection, which either leads to stronger differentiation at adaptive than neutral markers between populations within habitats (local adaptations to particular patches of similar habitat) or maintains similar genetic adaptations across populations within habitats (similar regimes of positive selection leading to general adaptation to particular habitat type, henceforth referred to as homogenizing selection), resulting in weaker differentiation at adaptive than neutral markers (Johnson & Munshi‐South, 2017).

Many studies based on neutral genetic markers (mostly microsatellites) provided strong empirical evidence for varying levels of differentiation between urban and nonurban bird populations, but these patterns were primarily attributed to drift (reviewed in Miles et al., 2019). In contrast, microevolutionary mechanisms underlying adaptive differentiation between urban and rural birds remain relatively little explored. Adaptations to urban environment are frequently polygenic, as recently shown by an emerging field of urban landscape genomics (Perrier et al., 2018). So far, genotype–phenotype association studies suggested that genes involved in neuronal processes and regulation of neural functions (e.g. neurotransmitter levels) may be at the forefront of behavioural adaptations to urban life (e.g. Mueller et al., 2013, 2020). For example, a significant enrichment of genes expressed in synapses and associated with neuron projections was found in the urban‐nonurban habitat association analysis in the burrowing owl *Athene cunicularia* (Mueller et al., 2020). Mueller et al. (2020) concluded that neuronal genes responsible for behavioural control functions may be a significant target of selection in novel urban environment. Other studies provided support for associations between single nucleotide polymorphisms (SNPs) in personality‐related genes (SERT, ADCYAP1 and DRD4) and urban‐nonurban phenotypes in birds (Mueller et al., 2013; Riyahi et al., 2017). van Dongen et al. (2015) found a relationship between SERT (but not DRD4) genotype and urbanization level in the black swan *Cygnus atratus*, suggesting that wariness in urban black swans may be genetically determined. However, these kinds of association analyses were mostly limited to a narrow range of model passerine species. Thus, it still remains unclear whether genetic adaptations to urban life are taxon‐specific or rather reflect general processes, and much wider phylogenetic coverage is necessary to draw robust conclusions.

Here, we used a non‐model bird species, the Eurasian coot *Fulica atra*, to investigate mechanisms of adaptation to urban environment. Eurasian coot is a common rail species (Rallidae, Gruiformes) naturally nesting on freshwater bodies and floodplains but undergoing a process of urbanization in central Europe over recent decades. Our previous behavioural studies in coots provided empirical support for the ‘urban wildlife syndrome’ hypothesis (Evans et al., 2010), showing elevated level of aggression and increased boldness in urban compared to nonurban individuals (Minias, 2015). However, the mechanisms underlying this behavioural differentiation (phenotypic plasticity vs. genetic adaptations) have not been investigated. In this study, we hypothesized that some behavioural differences between urban and nonurban coots may have a genetic background. To test this hypothesis, we sampled 160 adult coots from four pairs of urban and nonurban coot populations in Poland and examined associations between landscape urbanization level and polymorphisms in candidate genes previously reported to regulate circadian rhythm and key behavioural traits which may be important for adaptations to urban life. We also compared genetic differentiation of these behavioural genes and neutral markers (microsatellites) between urban and nonurban populations. We predicted that higher differentiation of behavioural genes (when compared to neutral microsatellites) between urban and nonurban populations would indicate a general genetic adaptation to urban environment, while higher differentiation in behavioural genes between particular urban populations would be consistent with the mechanism of local adaptations to particular urban sites.

## 2 MATERIALS AND METHODS

### 2.1 Study populations and sample collection

**FIGURE 1:**
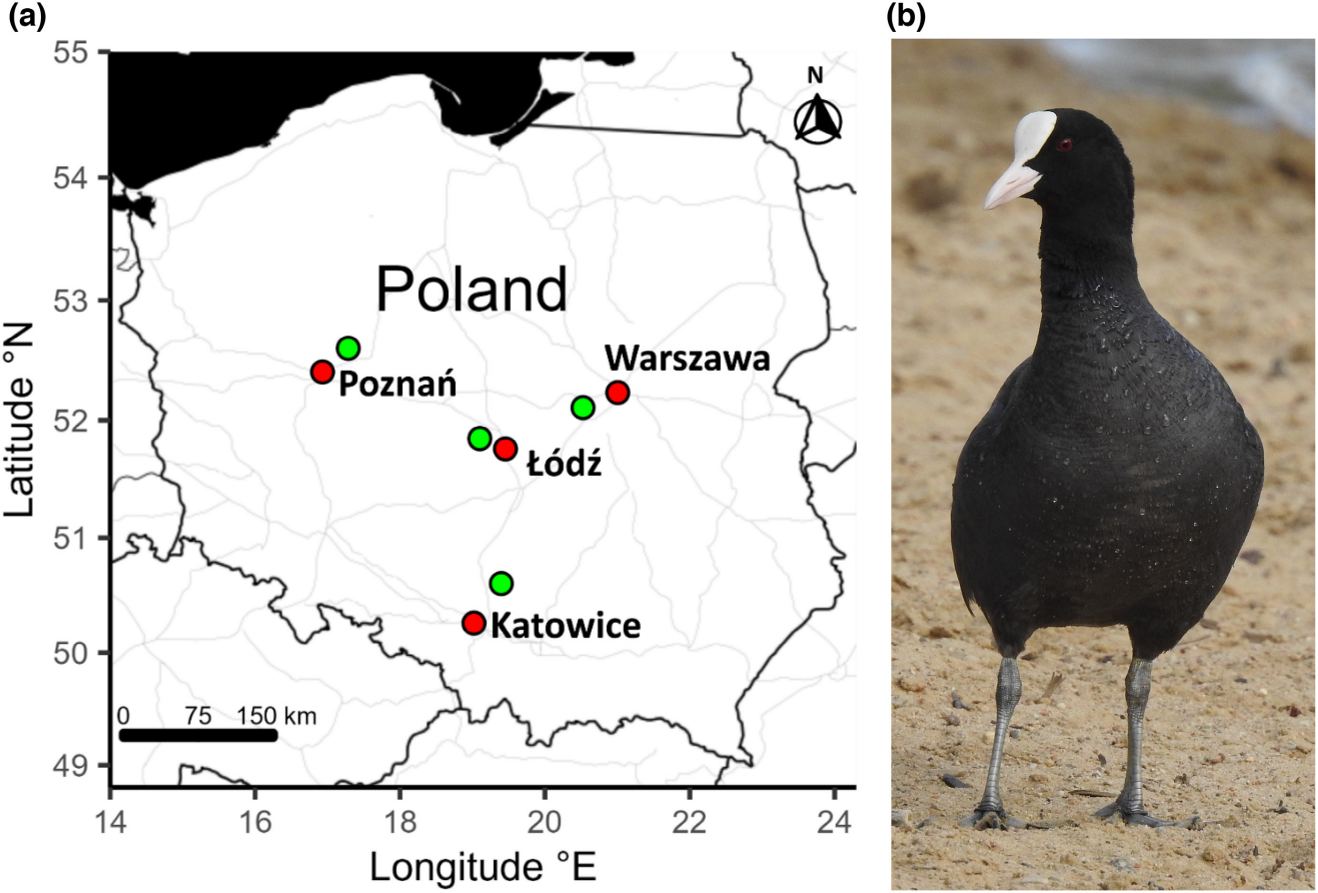
Location of paired urban (red) and nonurban (green) sampling sites of the Eurasian coot (a). The adult Eurasian coot (b).

Data were collected between 2009 and 2022 in four pairs of urban and nonurban coot populations from Poland. The study was performed in four urban agglomerations: Łódź (51°46′37″ N, 19°27′17″ E), Warsaw (52°13′48″ N, 21°00′40″ E), Poznań (52°24′30″ N, 16°56′01″ E) and Katowice (50°15′30″ N, 19°01′39″ E). Each agglomeration covered the area of >260 km^2^ and the number of inhabitants ranged from ca. 550 thousand (Poznań) to ca. 2.2 million (Katowice). The distance between the agglomerations ranged from 119 to 259 km. For each agglomeration, we chose a corresponding nearby nonurban site, so that the maximum distance between paired urban‐nonurban sites was <40 km (Figure 1). Nonurban sampling sites were located at the complexes of fish ponds (the average area of the complex = 0.58 km^2^) or natural fresh waterbodies and were characterized by low human disturbance, limited amount of noise and light pollution and the presence of extensive natural reed vegetation (over 50% water surface covered with vegetation, pers. obs.). All nonurban sampling sites were surrounded mainly by natural or semi‐natural areas (e.g. wetlands, agricultural areas, woodland, shrubs and wasteland), while the artificial areas covered less than ca. 5% of 2 km buffers around the nonurban sampling sites (QGIS. 3.32.2, QGIS Development Team 2023, Open Source Geospatial Foundation). In contrast, urban sampling sites were usually located in city centres with compact development, urban parks with high level of human pressure and other altered urban habitats often characterized by low availability of reed vegetation (<5% of water surface covered with vegetation, pers. obs.), thus providing limited protection to nests or chicks and enhancing alterations in behaviour. We captured 20 adult birds per population (*n* = 160 individuals in total) during the reproductive season (March–July). Birds were caught in noose traps made from monofilament line, mostly on nests while incubating (both urban and nonurban populations) or while feeding on the ground (only urban populations). All captured coots were individually marked with metal rings and plastic neck collars to avoid recaptures of the same individuals. Approximately 50 μL of blood was taken from the tarsal vein of each bird and stored in 96% ethanol at 5°C until DNA isolation. Genomic DNA was extracted using GeneMATRIX Tissue DNA Purification Kit (EURx).

### 2.2 Genotyping candidate behavioural genes

The DNA extracts were used to genotype 10 candidate genes, which were previously reported to associate with circadian rhythm (AANAT, CKIɛ, CKIδ, CLOCK, CREB1, NPAS2 and PERIOD2), migration (ADCYAP1), aggression (SERT) and exploratory behaviour (DRD4) in birds and other vertebrates (Holtmann et al., 2016; Steinmeyer et al., 2009). Following protocols developed by Steinmeyer et al. (2009), we mostly aimed to genotype either 3′UTR regions (ADCYAP1 and CREB1) or exonic regions (AANAT, CKIδ, CLOCK, NPAS2, PERIOD2). In CKIɛ gene, we genotyped two separate regions, including intron 2 with flanking part of exon 3 (CKIɛ_int2) and exon 5 with flanking intronic regions (CKIɛ_ex5). These regions were previously referred to as CKIɛ and CKIɛ‐*tau*, respectively (Steinmeyer et al., 2009). Following Holtmann et al. (2016) we also genotyped exonic regions in DRD4 and SERT genes. To develop amplification protocols for the Eurasian coot, we first tested original primers developed by Holtmann et al. (2016) and Steinmeyer et al. (2009). Although no genomic resources were available for our study species at the moment of primer screening, specificity of all primers was a posteriori verified using the recent de novo genome assembly of the Eurasian coot (GenBank: JABXFB010000017.1). The primers designed by Steinmeyer et al. (2009) were originally developed based on the combination of passerine (zebra finch *Taeniopygia guttata*) and non‐passerine (chicken *Gallus gallus* and wild turkey *Meleagris gallopavo*) sequences and, thus, they were expected to be well conserved across divergent avian lineages. In fact, the primers showed no mismatches with coot genome assembly within the 3‐terminus region, which is crucial for effective PCR amplification (Kwok et al., 1990). In contrast, the primers by Holtmann et al. (2016) were specifically designed for a single passerine species (dunnock *Prunella modularis*), which did not allow for effective cross‐application in the Eurasian coot. Consequently, we modified the primers for DRD4 exon 3 (DRD4_ex3‐F: 5′‐CTCCCGGCCGTTGATCTT‐3′ and DRD4_ex3‐R: 5′‐CTRAACTACAACCGGCGACA‐3′) and SERT exon 4 (SERT_ex4‐F: 5′‐CGCCAAGTTCTACAGGTGCA‐3′ and SERT_ex4‐R: 5′‐TGCCAGATGTTTTGACCCCT‐3′), using available genomic resources.

All PCR amplifications were carried out in a final volume of 20 μL containing 10 μL of DreamTaq PCR Master Mix (Thermo Fisher Scientific Inc.), 8 μL of nuclease‐free water, 0.5 μL of each primer at 10 μM concentration and 1 μL of DNA isolate. We used original PCR protocols developed by Holtmann et al. (2016) and Steinmeyer et al. (2009) for all markers except for the annealing temperature modifications in the amplification of DRD4 and SERT genes (both *T*_a_ = 53). The effects of PCR amplifications of all genes were confirmed by visual examination using 2% agarose gel electrophoresis and all genes, except AANAT, showed successful amplifications. Ad hoc modifications of PCR protocols and primers for AANAT were not successful and, thus, we excluded this gene from further analyses.

We used Sanger sequencing method to genotype the remaining nine candidate genes. We received high‐quality sequences for seven genes (CKIδ, CLOCK, CREB1, DRD4, NPAS2, PERIOD2, SERT) and for these genes we performed screening for single nucleotide polymorphisms (SNPs) using 10 randomly selected samples. We found evidence of polymorphism in only two genes (DRD4 and CREB1) and both of them were successfully sequenced across all sampled coots (*n* = 160). PCR products of DRD4 exon 3 (468 bp) and CREB1 3′UTR (531 bp) were sequenced in both forward and reverse directions. Sequences were assembled, trimmed to uniform lengths within each gene, and aligned using Geneious 10.0.5 software (Biomatters Ltd.). Unphased sequences of each gene were assigned to haplotypes using the PHASE algorithm in DnaSP v6.12.03 software (Rozas et al., 2017). Individuals homozygous at either gene were relatively frequent within our dataset (32% and 43% for CREB1 and DRD4, respectively), which enhanced reliable phasing.

To test whether unsuccessful Sanger sequencing of the remaining two genes (ADCYAP1 and CKIɛ) could be attributed to allele size variation resulting from the presence of genic microsatellites (Mueller et al., 2011), we used fragment size analysis for their genotyping. We followed the same amplification protocols as described above, except for the fluorescent labelling of forward primers with FAM. Fragment size analysis was conducted with ABI 3730XL capillary sequencer (Applied Biosystems). Allele sizes were scored against GeneScan TM 600 LIZ Standard (Applied Biosystems) in Geneious 10.0.5, and we found evidence for allele size polymorphism in ADCYAP1 3′UTR, CKIɛ intron 2 and CKIɛ exon 5. We successfully obtained information on allele sizes of these regions across all sampled coots (*n* = 160). To check for repeatability for allele size scoring, we repeated fragment size analysis for 30 randomly chosen samples (8–12 per locus), which were amplified in independent PCR runs. We found 100% repeatability in allele size scoring across all three markers. Both microsatellite alleles and phased haplotypes are henceforth referred to as allelic variants.

Our final analyses focused on four candidate gene showing polymorphism in the Eurasian coots: ADCYAP1, CKIɛ (CKIɛ_int2 and CKIɛ_ex5), CREB1 and DRD4. ADCYAP1 gene encodes pituitary adenylate cyclase‐activating polypeptide (PACAP), which functions as a neurotransmitter, neuromodulator and hypophysiotropic hormone involved in multiple biological processes, such as clock genes expression, melatonin production and energy metabolism (de Almeida et al., 2022; Mueller et al., 2011). So far, polymorphism of allele size in 3′UTR region of ADCYAP1 was reported to be associated with the level of landscape urbanization in the blackbird (Mueller et al., 2013). CKIɛ gene encodes casein kinase I (CKI) family enzymes responsible for phosphorylation of PER proteins (Eide et al., 2005). In humans, CKIɛ was reported to contain polymorphisms associated with delayed sleep phase syndrome (DSPS) (Takano et al., 2004). Also, a semidominant mutation in CKIɛ gene identified in Syrian hamsters *Mesocricetus auratus* was responsible for decreased kinase activity and shortened circadian rhythm (Lowrey et al., 2000). CREB1 gene encodes cAMP response element binding protein, which is a transcription factor mediating expression of CREB1‐dependent genes in various cell types. CREB1 protein is activated by phosphorylation process in response to multiple physiological stimuli (Lonze & Ginty, 2002). DRD4 gene encodes protein receptor activated by a catecholamine neurotransmitter—dopamine, involved in many functions of the central nervous system, such as learning, memory and motivation (Rondou et al., 2010).

### 2.3 Genotyping neutral microsatellites

All captured birds were genotyped at 15 non‐genic microsatellite loci to assess the level of neutral genetic variation in our study populations. We used markers originally designed for the Eurasian coot (Lv et al., 2017) and other rail species (Brackett et al., 2013; Buchan, 2000; Molecular Ecology Resources Primer Development Consortium, 2009). Microsatellite loci were amplified in multiplexes (3–4 marker per multiplex) using forward primers fluorescently labelled with FAM, VIC, ROX, and HEX. PCR amplifications were conducted in reaction mixtures containing 10 μL of QIAGEN Multiplex PCR Master Mix (QIAGEN), 1 μL of DNA template, 3 or 4 pairs of primers (0.2 μM of each primer) and a top‐up of nuclease‐free water to a final volume of 20 μL. Amplifications were carried out under the following conditions: 15 min at 95°C; 30 cycles consisting of 30 s at 94°C, 30 s at the annealing temperature, 60 s at 72°C; followed by 30 min of the final extension at 60°C. Fragment size analysis was conducted as described for genic microsatellites. To assess repeatability of allele scoring, we repeated amplifications and genotyping of 5% randomly chosen samples for each locus (120 samples in total). We found high repeatability (98.3%) in allele scoring between two independent genotyping runs. No evidence for linkage disequilibrium was found between any pairs of loci, as assessed in FSTAT 2.9.4 (Goudet, 1995). No deviations from Hardy–Weinberg equilibrium (HWE) were found (except for two loci deviating from HWE in a single population per locus), as assessed in GenAlEx 6.51b2 software (Peakall & Smouse, 2006, 2012; Table S1). *p* Values in these analyses were adjusted for multiple comparisons using the Bonferroni correction. The frequency of null alleles was low [0.0.19 ± 0.045 (SE)], as assessed in Cervus 3.0.7 software (Table S1). Finally, we found no evidence for genotyping errors due to stuttering or large allele dropout, as assessed in Micro‐Checker 2.2.3 (van Oosterhout et al., 2004).

### 2.4 Associations between genotypes and landscape urbanization level

To test for associations between genotypes (allelic variants and SNPs at candidate behavioural genes) and landscape urbanization level we used two different types of models, that is the additive and overdominant effect model. These models cover all potential additive and non‐additive allele effects, including dominant and recessive ones (Mueller et al., 2013). In additive models for SNPs, homozygotes of the minor allele were coded as 2, heterozygotes as 1 and homozygotes of the major allele as 0. In additive models for allelic variants, homozygotes of the analysed allelic variant were coded as 2, heterozygotes as 1 and the other genotypes as 0. In overdominant models, homozygotes of the major allele (or analysed allelic variant) were coded as 1 and the other genotypes were coded as 0. All these models were tested using the generalized linear mixed model (GLMM) approaches. In each model, binary landscape urbanization level (i.e. urban vs. nonurban landscape) was entered as a response variable and, thus, all the models were run for the binomial distribution and logit link function. Genotype was entered as either covariate (additive models) or fixed factor (overdominant models), while the identity of each population pair was entered as a random factor. The models were run only for allelic variants and SNPs with >3% frequency. All computations were performed in the *lme4* package (Bates et al., 2015) developed for the statistical environment R v.4.0.3 (R Foundation for Statistical Computing). The significance of all models was quantified with permutation tests (*n* permutations = 999) performed in the *predictmeans* R package (Welham et al., 2004). To address the problem of multiple comparisons, raw *p‐*values obtained for associations of landscape urbanization level with genotypes (i.e. candidate gene SNPs and haplotype/microsatellite allelic variants) were corrected for the false discovery rate (FDR) (Benjamini & Hochberg, 1995).

### 2.5 Genetic differentiation between populations

We used two different statistics to assess pairwise genetic differentiation between our study (urban and nonurban) populations. First, we computed *F*_ST_ values in GenAlEx software. Second, we used *strataG* R package (Archer et al., 2017) to calculate pairwise Jost's *D* statistics, which are independent from heterozygosity levels of compared populations (Jost, 2008). Statistical significance of both *F*_ST_ and Jost's *D* was assessed using permutation tests (*n* permutations = 1000) and all *p* values were corrected for FDR. Both statistics were calculated separately for each behavioural gene and across all neutral microsatellite loci. To assess whether population differentiation at adaptive markers (behavioural genes) was primarily explained by drift, we compared *F*_ST_ values calculated for each behavioural gene with *F*_ST_ values for neutral microsatellite markers. We expected that similar population differentiation between both types of markers would indicate that behavioural gene polymorphism was primarily shaped by drift, while stronger (or weaker) differentiation of adaptive than neutral markers would be due to selection at behavioural genes. These differences were tested separately for three different types of within‐ and between‐habitat comparisons: (i) nonurban vs. nonurban, (ii) nonurban vs. urban and (iii) urban vs. urban. Due to non‐normal distribution of *F*_ST_ values, we used nonparametric Friedman ANOVA test for dependent samples and pairwise post hoc Wilcoxon comparisons. We also aimed to compare population differentiation at adaptive markers within similar and between different habitats and, for this purpose, we tested for the differences in gene‐specific *F*_ST_ values between the three types of within‐ and between‐habitat comparisons listed above. Here, we used nonparametric one‐way Kruskal–Wallis test with post hoc comparisons. Since pairwise *F*_ST_ and Jost's *D* estimates were highly correlated and showed qualitatively and quantitatively similar patterns of population differentiation (Table S2), we reported only the results of *F*_ST_ analyses. All computations were performed in JMP 17.0 software (SAS Institute Inc.). All values are reported as means ± SE, unless otherwise stated.

## 3 RESULTS

### 3.1 Candidate gene polymorphism

We retrieved 13 CREB1 and 20 DRD4 allelic variants across all urban and nonurban populations. We identified fewer polymorphic sites and lower nucleotide diversity in CREB1 than DRD4, although haplotype diversity showed the opposite pattern (Table 1). ADCYAP1, CKIɛ_int2 and CKIɛ*_*ex5 were moderately polymorphic, showing between five and seven alleles per marker (Table 1). The mean observed heterozygosity at our candidate behavioural genes ranged from 0.44 ± 0.05 to 0.68 ± 0.02 and the mean expected heterozygosity ranged from 0.39 ± 0.04 to 0.67 ± 0.03 (Table 1).

**TABLE 1:** Genetic diversity measures for five regions in four behavioural genes.

Gene/region	*N*_A_	Ho (mean ± SE)	He (mean ± SE)	*S*	Hd (mean ± SD)	π (mean ± SE)
CREB1	13	0.68 ± 0.02	0.65 ± 0.02	10	0.67 ± 0.02	0.0020 ± 0.0001
DRD4	20	0.58 ± 0.04	0.57 ± 0.02	16	0.59 ± 0.03	0.0034 ± 0.0003
ADCYAP1	7	0.68 ± 0.05	0.67 ± 0.03	NA	NA	NA
CKIɛ_int2	7	0.44 ± 0.05	0.39 ± 0.04	NA	NA	NA
CKIɛ_ex5	5	0.59 ± 0.04	0.56 ± 0.01	NA	NA	NA

Abbreviations: Hd, haplotype diversity; He, expected heterozygosity; Ho, observed heterozygosity; *N*_A_, number of allelic variants; S, number of polymorphic sites; π, nucleotide diversity.

### 3.2 Genotype–landscape associations

**FIGURE 2:**
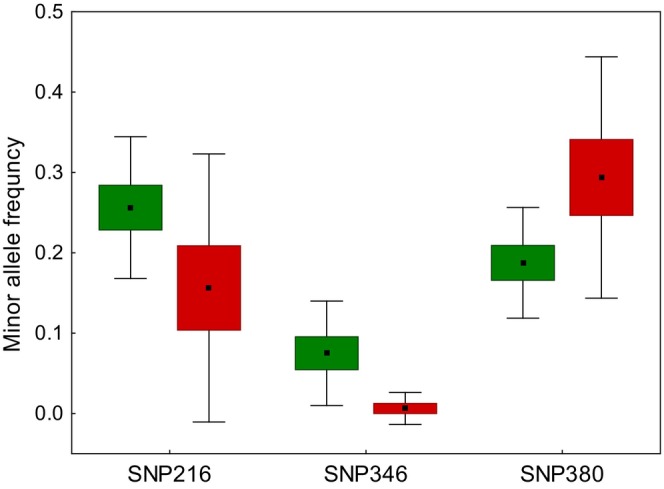
Mean frequency of the minor allele in three SNPs of CREB1 gene. Means (central points), SE (box) and 95% confidence intervals (whiskers) of allele frequency are shown for urban (red) and nonurban (green) populations.

Additive models revealed significant associations between the level of habitat urbanization and three out of four tested SNP genotypes in CREB1 (Figure 2). Two of these SNPs showed higher frequency of the major allele in urban than nonurban populations (SNP216 and SNP346), whereas the third SNP showed the opposite pattern with the frequency of the major allele being higher in nonurban populations (SNP380) (Figure S1 and Table 2). We also found two significant associations between the urbanization level and inferred CREB1 haplotypes. Specifically, CREB*02 and CREB*04 haplotypes had higher and lower frequency in urban populations, respectively (Figure S2 and Table 2). In fact, CREB*04 was the most common haplotype which contained the minor allele at SNP346 (Figures S1B, S2B). Statistical significance of all genotype–landscape associations revealed by additive models for CREB1 was retained after correction for multiple comparisons (FDR) and was supported by permutation tests (all *p* < .05). Overdominant models showed only two significant associations for CREB1 (SNP346 and CREB1*04 haplotype), but they both lost significance after FDR correction (Table 2). No significant associations were detected by either additive or overdominant models for SNP genotypes (*n* = 2) and haplotypes (*n* = 5) at DRD4 gene (all *p* > .05; Table S3). Similarly, no significant genotype–landscape associations were found for ADCYAP1, CKI_int2 and CKIɛ_ex5 allelic variants (total number of tested variants: *n* = 10, all *p* > .05; Table S4).

**TABLE 2:** Associations between CREB1 3′UTR genotypes and landscape urbanization level in eight paired urban and nonurban populations of the Eurasian coot.

Genotype	Variant	Additive model			Overdominant model		
		Coefficients (mean ± SE)	*Z*	*p*	Coefficients (mean ± SE)	*Z*	*p*
Haplotype	CREB1*01	−0.02 ± 0.22	−0.11	.91	0.35 ± 0.32	1.11	.27
	CREB1*02	**0.72 ± 0.29**	**2.47**	.014*	0.54 ± 0.33	1.63	.104
	CREB1*03	−0.21 ± 0.33	−0.65	.52	0.00 ± 0.35	0.00	1.00
	CREB1*04	**−2.53 ± 1.06**	**−2.40**	.017*	**−2.53 ± 1.06**	**−2.40**	**.017**
SNP	**CREB1_SNP216**	**−0.68 ± 0.30**	**−2.26**	.024*	−0.55 ± 0.34	−1.65	.099
	**CREB1_SNP346**	**−2.63 ± 1.05**	**−2.50**	.012*	**−2.63 ± 1.05**	**−2.50**	**.012**
	**CREB1_SNP380**	**0.63 ± 0.28**	**2.24**	.025*	0.48 ± 0.33	1.46	.14
	CREB1_SNP393	0.00 ± 0.60	0.00	1.00	0.00 ± 0.60	0.00	1.00

*Note*: Associations were tested for the most common haplotypes (*n* = 4) and single nucleotide polymorphisms (SNP) (*n* = 4) using additive and overdominant models. Population pair was used a as random factor. Significant associations (as inferred based on uncorrected *p*‐values) were bolded. Associations which retained statistical significance after FDR correction were indicated with asterisks (*).

### 3.3 Genetic differentiation between populations

**FIGURE 3:**
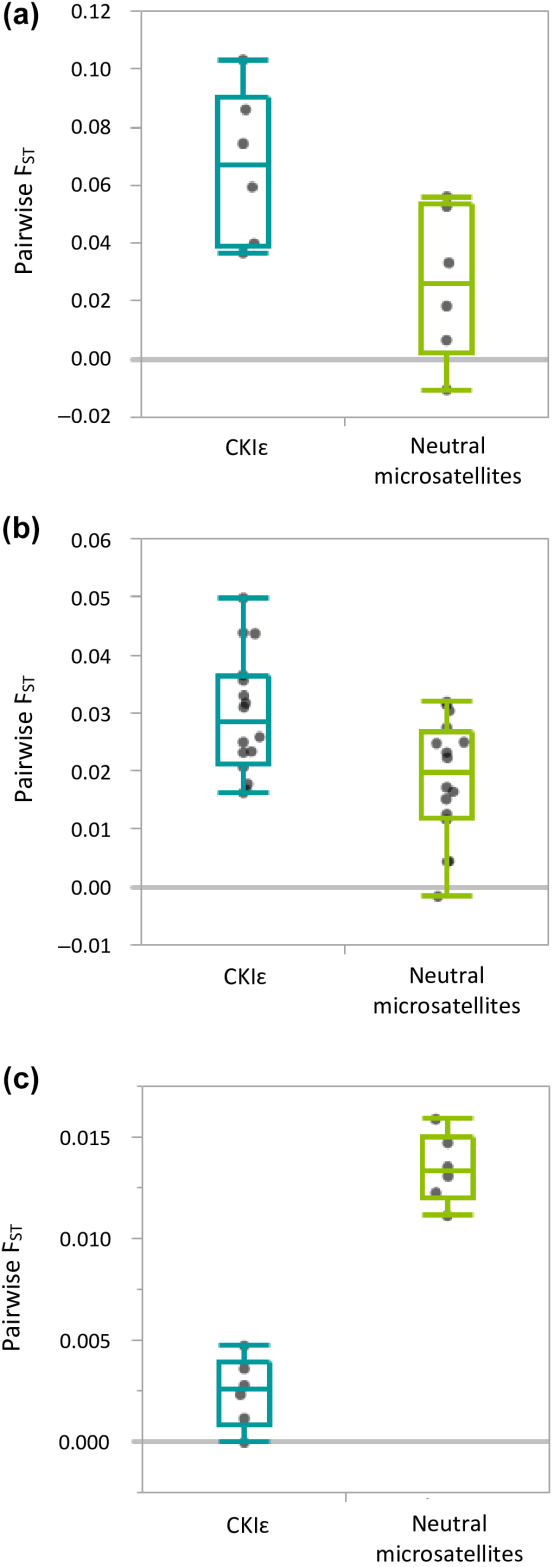
Pairwise population differentiation (*F*_ST_) for CKIɛ_int2 and neutral microsatellites, as assessed among urban populations (a), between nonurban and urban populations (b) and among nonurban populations (c). Medians (central point), Q1 and Q3 quartiles (box) and range values (whiskers) are shown.

Genetic differentiation between our study populations was most apparent at CKIɛ_int2 region. We found that differentiation at CKIɛ_int2 was significantly lower among nonurban populations, when compared to differentiation among urban populations (*Z* = 2.65, *p* = .008), as well as between nonurban vs. urban populations (*Z* = 2.70, *p* = .007). We also found that differentiation among urban populations was significantly higher at CKIɛ_int2, when compared to neutral microsatellite markers (χ^2^ = 5.03, *p* = .025; Figure 3a). Similarly, higher differentiation was found between urban vs. nonurban populations at CKIɛ_int2 than neutral microsatellites (χ^2^ = 6.57, *p* = .010; Figure 3b). The opposite pattern was found for differentiation among nonurban populations, which was significantly lower at CKIɛ_int2 than neutral microsatellites (χ^2^ = 8.31, *p* = .004; Figure 3c). Finally, CKIɛ_int2 showed the largest number of significant pairwise *F*_ST_ values (*n* = 7), although only two of them retained significance after FDR correction (Table 3). Notably, pairwise *F*_ST_ values at CKIɛ_int2 were significant only for urban–urban and urban‐nonurban comparisons, while all pairwise comparisons among nonurban populations were nonsignificant (Table 3).

**TABLE 3:** Population differentiation at two regions of behavioural genes (CKIɛ and CREB1 3′UTR) between urban (Urb) and nonurban (NonUrb) populations of the Eurasian coot, as measured with *F*_ST_ (below diagonal) and Jost's *D* (above diagonal) values.

Gene/region	Sampling location	Landscape	Łódź		Poznań		Katowice		Warszawa	
			NonUrb	Urb	NonUrb	Urb	NonUrb	Urb	NonUrb	Urb
CKIɛ_int2	Łódź	NonUrb	–	0	0	0.027	0	0	0	0.055
		Urb	0.012	–	0	**0.073**	0	0.023	0	0.009
	Poznań	NonUrb	0.007	0.001	–	0.041	0	0.004	0	0.039
		Urb	0.031	**0.077**	**0.064**	–	0.041	0.001	0.016	0.194*
	Katowice	NonUrb	0.003	0.003	0.001	**0.052**	–	0.004	0	0.039
		Urb	0.016	**0.053**	0.042	0.004	0.032	–	0	**0.115**
	Warszawa	NonUrb	0.003	0.003	0.001	**0.052**	0.000	0.032	–	**0.073**
		Urb	0.050	0.016	0.023	0.138*	0.030	0.111*	0.030	–
CREB1	Łódź	NonUrb	–	0.030	0.000	0.019	0	0.015	0.016	**0.150**
		Urb	0.017	–	0.024	0.017	0	**0.103**	**0.097**	0.046
	Poznań	NonUrb	0.004	0.015	–	0.021	0	0.012	0.018	**0.163**
		Urb	0.017	0.013	0.016	–	0	0	0	0.025
	Katowice	NonUrb	0.009	0.007	0.007	0.004	–	0.006	0	0.023
		Urb	0.014	0.027	0.015	0.007	0.012	–	0	**0.131**
	Warszawa	NonUrb	0.011	0.021	0.011	0.009	0.012	0.003	–	**0.103**
		Urb	**0.039**	0.024	**0.037**	0.019	0.018	**0.045**	**0.047**	–

*Note*: Significant associations (as inferred based on uncorrected *p‐*values) were bolded. Associations which retained statistical significance after FDR correction were indicated with asterisks (*).

Similarly to CKIɛ_int2, genetic differentiation at CREB1 was significantly higher among urban populations (*Z* = 2.16, *p* = .031), as well as between urban and nonurban populations (*Z* = 2.17, *p* = .030), than among nonurban ones. Despite this pattern, we found no significant difference in population differentiation between CREB1 and neutral microsatellites for urban‐nonurban and urban–urban comparisons (all *p* > .05), while population differentiation among nonurban populations was higher at neutral microsatellites than CREB1 (χ^2^ = 5.77, *p* = .016). We identified four significant *F*_ST_ for CREB1, although all values lost significance after FDR correction (Table 3).

There were no differences in *F*_ST_ values between different types of comparisons (within and between habitats) at all the remaining adaptive markers (ADCYAP1, CKIɛ_ex5, and DRD4) (all *p* > .05). Also, population differentiation at these markers was significantly lower when compared to neutral microsatellites, although this pattern was apparent only in some of the comparisons, mostly among nonurban populations (ADCYAP1 and CKIɛ_ex5) and between urban and nonurban populations (CKIɛ_ex5 and DRD4) (Table S5). No significant *F*_ST_ values were identified for DRD4, while all pairwise *F*_ST_ for ADCYAP1 and CKI_ex5 lost significance after FDR correction (Table S2).

Genetic differentiation at neutral microsatellites between urban vs. nonurban populations was significantly lower than among urban populations (*Z* = 2.54, *p* = .011), but significantly higher than among nonurban ones (*Z* = 2.03, *p* = .043). Many pairwise *F*_ST_ and Jost's *D* values were significant for neutral microsatellites (*n* = 30), but in contrast to behavioural genes, significant neutral differentiation was not only apparent in urban–urban and urban‐nonurban comparisons, but also among different nonurban populations (Table S2).

## 4 DISCUSSION

Our study provided empirical evidence for genetic basis of adaptation (both local and general) to urban environment in the Eurasian coot. We found consistent associations between polymorphisms in 3′UTR region of CREB1 gene and the level of landscape urbanization across four pairs of urban‐nonurban coot populations. We also identified two CREB1 haplotypes, which significantly differed in frequency between urban (higher CREB*02 frequency) and nonurban (higher CREB*04 frequency) populations. Finally, we showed that genetic differentiation at CKIɛ intronic region between urban and nonurban populations was significantly stronger than differentiation at neutral microsatellite markers and there was even stronger differentiation at CKIɛ intron among different urban populations. Although we lacked evidence for linkage of this intronic variation with coding polymorphisms within CKIɛ gene, the results suggest possible local adaptation in CKIɛ expression regulation to particular urban sites.

In general, CREB1 gene codes for transcription factor protein not only involved in multiple cellular processes in nervous tissue, such as neurogenesis, neuronal differentiation, neuroprotection and synaptic plasticity, but also responsible for cognitive functions, such as learning and long‐term memory formation (Alberini, 2009; Silva et al., 1998). Multifunctional character of CREB1 gene was demonstrated in a wide range of studies conducted across diverse animal groups, including insects, molluscs and mammals (reviewed in Silva et al., 1998). However, the role of CREB1 expression is relatively little explored in birds and this kind of research was primarily limited to songbirds (Passeriformes), in which learning and memory are crucial in the development of vocal abilities (Bolhuis & Gahr, 2006). For instance, CREB1 protein phosphorylation level in the high vocal centre (HVC) in brain affected processes of creating the long‐term memories in zebra finches and song sparrows *Melospiza melodia* (Reeves, 2005; Sakaguchi et al., 1999). Similarly, reduced CREB1 activity had a negative influence on the process of postnatal vocal learning and development of conspecific song memory in transgenic zebra finches (Abe et al., 2015). A crucial role of cognitive traits in urbanization processes was confirmed in several avian behavioural studies showing that urban birds are usually more explorative, better in problem solving and learning, and have better long‐term memory retention (Audet et al., 2016; Kozlovsky et al., 2017). Combination of these traits may facilitate recognition of unfamiliar food sources, implementation of alternative foraging strategies and habituation to elevated human disturbance (Lee & Thornton, 2021; Szulkin et al., 2020). Elevated perception, more exploratory behaviour and creating long‐term memories could be especially beneficial during the breeding season, when multiple circumstances and choices may decide about reproductive success or failure (Németh et al., 2017; Reeves, 2005). Our previous studies in the Eurasian coot indicated that urban individuals are more willing to exploit novel anthropogenic food sources and show elevated boldness towards humans when compared with nonurban conspecifics (Minias et al., 2018). We also found a significant positive relationship between territory occupancy and reproductive success in urban coots, suggesting that they may have capabilities to reliably assess territory quality in a novel urban environment (Minias & Janiszewski, 2016). These patterns should likely be attributed to better cognition and exploratory behaviour, which may be linked to genetic differences in the expression of key behavioural genes. Cognition also allows birds to gather information about environmental conditions and, then, to use the acquired knowledge in decision‐making processes, which is crucial to properly match the choice of local environment to phenotype (Szulkin et al., 2020). Our previous study showed that landscape selection (in terms of urbanization level) is highly consistent over the annual cycle in coots, despite migrating at relatively long distances (usually hundreds km or more; Chyb et al., 2021). This can be explained by the matching habitat choice hypothesis, assuming that birds select habitats, to which they are best physiologically and behaviourally adapted (Edelaar et al., 2008). The consistency in habitat choice may be heritable and possibly result from mutations in transcription factors of key genes responsible for cognitive functions, such as CREB1. However, we acknowledge that non‐genetic mechanisms (e.g. natal habitat preferences induction, NHPI) may also contribute to consistency in habitat selection.

CREB1 gene is also predicted to be involved in modulation of several non‐cognitive behavioural traits and physiological processes, which may shape the way birds interact with novel (urban) environment and respond to novel stressors. For instance, CREB1 allele size was related to dispersal phenology in the common buzzard *Buteo buteo* (Chakarov et al., 2013) and to dispersal propensity in South Pacific populations of silvereye *Zosterops lateralis* (Estandia et al., 2023). Identification of two cAMP response element binding protein sites in a potential promoter region of chicken ghrelin gene suggests that CREB1 protein may be one of the transcription factors affecting food intake, growth hormone release, energy balance and level of corticosterone via coregulation of ghrelin secretion (Richards et al., 2006). CREB1 is also a candidate gene for coregulation of circadian rhythm in birds (Steinmeyer et al., 2009) and other vertebrates (e.g. Asher & Schibler, 2011). Light indirectly increases the level of CREB1 protein phosphorylation via suppressing melatonin secretion (Bentley, 2001). Nevertheless, few studies showed relationships between CREB1 polymorphism and clock‐dependent traits in birds. CREB1 allele size was associated with the length of the incubation period in male tree swallows *Tachycineta bicolor* (Bourret & Garant, 2015) and moult rate in the willow warbler *Phylloscopus trochilus* (Bazzi et al., 2017). During a long‐term monitoring of Eurasian coots, we observed that several reproductive parameters showed conspicuous variation along the urban‐nonurban gradient (Minias, 2016). Although quantitative data on phenological variation was not subject to any formal analysis, casual observations indicate that urban coots often initiate breeding season earlier than nonurban individuals, which follows earlier melting of water ice cover in urbanized landscape (PM, pers. obs.). In fact, a wide range of studies showed advanced reproductive phenology and altered circadian activity patterns in urban bird populations, which may be triggered by elongated photoperiod in highly urbanized areas (reviewed in Deviche & Davies, 2014). Associations between CREB1 polymorphism and breeding phenology in urban and nonurban coots (as well as other avian species) should be put to further investigation.

While the analyses of CREB1 3′UTR showed evidence for general adaptations to urban landscape across different pairs of urban‐nonurban populations, genetic differentiation at CKIɛ_int2 was rather consistent with the mechanism of local adaptations. Selective landscape may show a considerable variation between different urban agglomerations enhanced by a complex structure of urban habitats. Specific urban sites may be associated with different optima of behavioural trait expression, which may ultimately result in local adaptations at the genetic level. In our study, both CREB1 and CKIɛ_int2 showed stronger differentiation between urban and nonurban populations, when compared to differentiation among nonurban populations. We also found strong differentiation among urban populations at these genes, but only differentiation at CKIɛ_int2 was stronger than differentiation at neutral microsatellite markers, suggesting it may not be exclusively attributed to drift. Instead, strong differentiation among urban populations at CKIɛ_int2 may be driven by natural selection acting at linked coding (exonic) regions. Intronic polymorphisms may be tightly linked with adaptive exonic variation within haplotypes, but intronic repeatable elements were also found to be involved in coregulation of gene expression via multiple molecular processes, such as gene transcription, splicing or RNA export to the cytoplasm (Li et al., 2004). Although the patterns of population differentiation at CKIɛ_int2 suggest local adaptations at either linked coding regions or intron‐mediated gene expression regulation, the exact mechanism underlying this variation still needs to be determined. In contrast to CKIɛ_int2, significantly lower differentiation among nonurban populations was found in most of our candidate genes (when compared to neutral microsatellites markers), which may be due to homogenizing selection. The maintenance of the same genetic adaptations across different nonurban populations suggests that environmental conditions are relatively similar across the nonurban landscape.

We have found no evidence for general or local associations between landscape urbanization level and polymorphisms in ADCYAP1 and DRD4. In contrast to our results, previous studies on blackbirds revealed relationships of habitat urbanization level with polymorphisms at both of these genes, although they were relatively weak (Mueller et al., 2013). ADCYAP1 was also reported to associate with migratory propensity, distance and restlessness in birds (de Almeida et al., 2022; Mueller et al., 2011). Similarly, relationships between DRD4 gene polymorphism and exploratory behaviour were found in several passerine bird species (e. g. Garamszegi et al., 2014; Mueller et al., 2014). Despite no evidence for significant genotype–landscape associations in ADCYAP1 3′UTR, our results suggest that polymorphisms in behavioural genes are likely to prevail in non‐coding regions (e.g. CREB1 3′UTR and CKIɛ_int2) which may co‐regulate gene expression, than in coding gene regions, where non‐silent mutations introduce alterations in protein structure. This conclusion is consistent with the recent findings based on transcriptomic analyses, where regulation of gene expression was identified as one of the key mechanism responsible for adaptations of animals to urban life (Harris et al., 2015; Watson et al., 2021).

Although our analyses provided convincing evidence for genetic differentiation at behavioural genes between urban and nonurban coot populations, we need to acknowledge some limitations of our approach. To determine the baseline neutral level of genetic differentiation between our study populations, we used a set of randomly selected microsatellite loci. In general, microsatellites are considered a versatile and robust tool used to describe neutral evolutionary processes in a wide range of population genetics and molecular ecology studies (e.g. Hartmann et al., 2014; Perrin et al., 2021). However, a growing body of evidence contests the pure neutral character of microsatellites (due to their possible location in coding regions or linkage with adaptive markers, which may lead to genetic hitchhiking) and identify putative functional roles of microsatellite DNA (e.g. in regulation of gene expression) (Li et al., 2004; Selkoe & Toonen, 2006). Taking this into account, we conclude that neutrality of microsatellites should not be taken for granted and interpretations of our results need to be cautious.

In conclusion, our study shows conspicuous associations between CREB1 genotypes and the level of landscape urbanization across central European populations of the Eurasian coot. At the same time, we recorded strong differentiation at CKIɛ intronic region among urban and nonurban populations, but also among particular urban sites. Our results suggest that behavioural differentiation between urban and nonurban individuals may have, at least partially, a heritable component and may result from microevolutionary processes. We conclude that specific environmental conditions in human‐dominated areas may create an effective environmental barrier for gene flow between urban and nonurban populations, enhancing both local and general adaptations to urban habitats. However, the complexity and multifaceted character of behavioural responses to novel environmental stimuli underpins the need for further investigation of personality‐related candidate genes in the context of urbanization processes across a wide spectrum of phylogenetically diverse taxa.

## AUTHOR CONTRIBUTIONS

**Amelia Chyb:** Conceptualization (equal); data curation (lead); formal analysis (lead); investigation (lead); methodology (lead); writing – original draft (lead); writing – review and editing (equal). **Radosław Włodarczyk:** Investigation (supporting); writing – review and editing (equal). **Joanna Drzewińska‐Chańko:** Investigation (supporting); writing – review and editing (equal). **Jan Jedlikowski:** Investigation (supporting); writing – review and editing (equal). **Kimberly K. O. Walden:** Formal analysis (supporting); writing – review and editing (equal). **Piotr Minias:** Conceptualization (equal); formal analysis (supporting); funding acquisition (lead); investigation (supporting); writing – review and editing (equal).

## FUNDING INFORMATION

The study was financially supported by the research grant of the National Science Centre in Poland (2020/38/E/NZ8/00143).

## CONFLICT OF INTEREST STATEMENT

The authors declare no competing interests.

## Supporting information


Appendix S1:

## Data Availability

All sequences generated and used in this study have been deposited in GenBank (Nos: OR605431‐OR605463). Raw data were attached as supporting material: Appendix S1.
