# Supplementary material for: Urbanization is associated with non‐coding polymorphisms in candidate behavioural genes in the Eurasian coot
Source: Ecol Evol. 2023 Oct 1;13(10):e10572. doi: 10.1002/ece3.10572 (PMC10542476; doi:10.1002/ece3.10572)
Supplement: Supplementary file 1 — Appendix S1: [file ECE3-13-e10572-s001.zip › ece310572-sup-0001-AppendixS1.pdf]

Appendix 1

Urbanization is associated with non-coding polymorphisms in candidate behavioural genes in the Eurasian coot

Amelia Chyb[1], Radosław Włodarczyk[1], Joanna Drzewińska-Chańko[1], Jan Jedlikowski[2], Kimberly K. O. Walden[3], Piotr Minias[1]

1. Department of Biodiversity Studies and Bioeducation, Faculty of Biology and Environmental Protection, University of Łódź, Banacha 1/3, 90-237, Łódź, Poland
2. Faculty of Biology, Biological and Chemical Research Centre, University of Warsaw, Żwirki i Wigury 101, 02-089, Warsaw, Poland
3. Roy J. Carver Biotechnology Center, University of Illinois at Urbana-Champaign, 1206 West Gregory Drive, Urbana, IL 61801, USA.

Correspondence and requests for materials should be addressed to P.M. (email: pminias@op.pl) and A.C. (email: chybamelia@gmail.com)

Table S1. Genetic diversity measures at fifteen mictrosatellite loci in four pairs of urban and nonurban populations of the Eurasian coot (N_A – number of alleles; He – expected heterozygosity; Ho – observed heterozygosity; F_{IS} – coefficient of inbreeding; χ^2 – results of Chi square test for Hardy-Weinberg disequilibrium; F_{null} – null alleles frequency)

Locus	Population	N_A	Ho	He	F_{IS}	Df	χ^2	P	F_{null}
Fa1	Łódź_urban	8	0.75	0.79	0.048	28	32.94	0.238	-0.001
	Łódź_nonurban	7	0.60	0.70	0.150	21	14.46	0.849	
	Poznań_urban	9	0.75	0.70	-0.077	36	34.29	0.550	
	Poznań_nonurban	8	0.75	0.74	-0.010	28	35.40	0.159	
	Śląsk_urban	7	0.60	0.63	0.040	21	14.35	0.854	
	Śląsk_nonurban	6	0.80	0.70	-0.150	15	5.63	0.985	
	Warszawa_urban	8	0.70	0.69	-0.018	28	42.36	0.040	
	Warszawa_nonurban	7	0.65	0.72	0.099	21	47.28	0.0009	
Fa2	Łódź_urban	13	0.85	0.83	-0.021	78	95.73	0.084	0.0025
	Łódź_nonurban	12	0.85	0.76	-0.110	66	46.54	0.967	
	Poznań_urban	17	0.95	0.90	-0.060	136	118.42	0.859	
	Poznań_nonurban	12	0.85	0.88	0.029	66	70.58	0.327	
	Śląsk_urban	10	0.85	0.85	0.004	45	51.63	0.231	
	Śląsk_nonurban	12	0.85	0.81	-0.053	66	72.80	0.264	
	Warszawa_urban	16	0.90	0.85	-0.056	120	111.72	0.693	
	Warszawa_nonurban	12	0.85	0.88	0.038	66	106.98	0.001	
Fa3	Łódź_urban	9	1.00	0.84	-0.190	36	38.390	0.362	0.0046
	Łódź_nonurban	8	0.75	0.76	0.010	28	24.96	0.630	
	Poznań_urban	10	0.90	0.80	-0.130	45	28.79	0.971	
	Poznań_nonurban	10	1.00	0.84	-0.190	45	52.84	0.197	
	Śląsk_urban	10	0.90	0.84	-0.078	45	70.42	0.009	
	Śląsk_nonurban	10	0.90	0.80	-0.130	45	27.82	0.979	
	Warszawa_urban	14	1.00	0.88	-0.130	91	82.83	0.717	
	Warszawa_nonurban	6	0.75	0.79	0.050	15	15.62	0.408	
Fa9	Łódź_urban	13	0.90	0.83	-0.084	78	76.12	0.539	0.0814
	Łódź_nonurban	9	0.75	0.78	0.037	36	17.56	0.996	
	Poznań_urban	13	0.90	0.85	-0.057	78	79.73	0.424	
	Poznań_nonurban	11	0.80	0.84	0.048	55	88.01	0.003	
	Śląsk_urban	12	0.75	0.86	0.130	66	68.34	0.398	

	Śląsk_nonurban	14	0.90	0.86	-0.042	91	93.21	0.416	
	Warszawa_urban	11	0.85	0.86	0.013	55	73.69	0.047	
	Warszawa_nonurban	14	0.58	0.89	0.350	91	124.64	0.011	
Fa10	Łódź_urban	11	0.85	0.88	0.033	55	50.45	0.649	0.0753
	Łódź_nonurban	12	0.90	0.88	-0.027	66	88.67	0.033	
	Poznań_urban	14	0.85	0.90	0.050	91	102.45	0.194	
	Poznań_nonurban	13	0.85	0.90	0.058	78	83.91	0.303	
	Śląsk_urban	11	0.45	0.83	0.460	55	91.74	0.001	
	Śląsk_nonurban	9	0.60	0.81	0.260	36	40.91	0.264	
	Warszawa_urban	12	0.80	0.85	0.055	66	65.15	0.506	
	Warszawa_nonurban	10	0.80	0.86	0.072	45	36.36	0.817	
Fa11	Łódź_urban	11	0.80	0.86	0.066	55	45.83	0.806	-0.0629
	Łódź_nonurban	10	0.85	0.88	0.030	45	54.26	0.162	
	Poznań_urban	12	0.90	0.90	0.000	66	68.31	0.399	
	Poznań_nonurban	14	0.80	0.88	0.090	91	98.41	0.279	
	Śląsk_urban	12	0.95	0.85	-0.120	66	45.56	0.974	
	Śląsk_nonurban	10	0.75	0.85	0.110	45	57.88	0.094	
	Warszawa_urban	9	0.85	0.84	-0.010	36	30.08	0.746	
	Warszawa_nonurban	9	0.50	0.77	0.350	36	85.89	0.000006	
Fa13	Łódź_urban	6	0.50	0.54	0.070	15	4.48	0.996	0.0084
	Łódź_nonurban	4	0.60	0.54	-0.100	6	2.92	0.819	
	Poznań_urban	7	0.75	0.63	-0.190	21	6.51	0.999	
	Poznań_nonurban	4	0.45	0.47	0.040	6	1.17	0.978	
	Śląsk_urban	5	0.60	0.51	-0.190	10	7.76	0.652	
	Śląsk_nonurban	4	0.40	0.38	-0.060	6	1.79	0.938	
	Warszawa_urban	5	0.65	0.61	-0.068	10	6.65	0.758	
	Warszawa_nonurban	3	0.45	0.45	0.008	3	0.29	0.961	
Fa14	Łódź_urban	16	0.60	0.92	0.346	120	154.92	0.017	0.0175
	Łódź_nonurban	15	0.70	0.91	0.234	105	130.64	0.046	
	Poznań_urban	18	0.85	0.92	0.079	153	132.67	0.881	
	Poznań_nonurban	19	0.90	0.92	0.016	171	208.89	0.026	
	Śląsk_urban	16	0.80	0.92	0.126	120	136.11	0.149	
	Śląsk_nonurban	13	0.75	0.90	0.163	78	97.59	0.066	

	Warszawa_urban	18	0.75	0.92	0.184	153	165.36	0.234	
	Warszawa_nonurban	11	0.70	0.84	0.163	55	67.36	0.122	
Fa15	Łódź_urban	4	0.45	0.61	0.258	6	8.51	0.203	0.0557
	Łódź_nonurban	4	0.45	0.54	0.165	6	5.95	0.429	
	Poznań_urban	5	0.60	0.53	-0.135	10	6.29	0.790	
	Poznań_nonurban	4	0.70	0.70	-0.005	6	5.77	0.450	
	Śląsk_urban	5	0.55	0.69	0.204	10	32.42	0.0003	
	Śląsk_nonurban	7	0.65	0.61	-0.059	21	6.31	0.999	
	Warszawa_urban	5	0.50	0.69	0.275	10	7.76	0.653	
	Warszawa_nonurban	4	0.50	0.58	0.143	6	4.20	0.649	
Fa16	Łódź_urban	5	0.55	0.57	0.027	10	4.23	0.936	0.0098
	Łódź_nonurban	4	0.70	0.56	-0.258	6	4.74	0.578	
	Poznań_urban	4	0.55	0.52	-0.065	6	10.40	0.109	
	Poznań_nonurban	4	0.45	0.40	-0.120	6	5.99	0.424	
	Śląsk_urban	5	0.65	0.53	-0.226	10	4.64	0.914	
	Śląsk_nonurban	5	0.70	0.56	-0.256	10	4.61	0.916	
	Warszawa_urban	6	0.65	0.54	-0.198	15	11.03	0.750	
	Warszawa_nonurban	4	0.45	0.51	0.120	6	13.71	0.033	
KiRa9	Łódź_urban	5	0.60	0.57	-0.055	10	1.66	0.998	-0.0199
	Łódź_nonurban	4	0.60	0.52	-0.151	6	5.42	0.491	
	Poznań_urban	5	0.40	0.44	0.091	10	20.14	0.028	
	Poznań_nonurban	4	0.40	0.46	0.137	6	5.03	0.541	
	Śląsk_urban	5	0.50	0.50	0.005	10	1.63	0.998	
	Śląsk_nonurban	4	0.45	0.48	0.060	6	1.87	0.932	
	Warszawa_urban	4	0.55	0.49	-0.125	6	12.39	0.054	
	Warszawa_nonurban	3	0.40	0.40	0.003	3	1.15	0.765	
KiRa10	Łódź_urban	9	0.90	0.77	-0.163	36	27.74	0.836	0.0405
	Łódź_nonurban	8	0.75	0.70	-0.071	28	21.52	0.803	
	Poznań_urban	11	0.70	0.65	-0.083	55	87.40	0.0035	
	Poznań_nonurban	7	0.75	0.73	-0.026	21	11.29	0.957	
	Śląsk_urban	8	0.60	0.78	0.233	28	24.47	0.657	
	Śląsk_nonurban	10	0.80	0.79	-0.019	45	65.14	0.026	
	Warszawa_urban	8	0.80	0.72	-0.111	28	26.54	0.543	

	Warszawa_nonurban	6	0.80	0.72	-0.120	15	8.76	0.890	
KiRa16	Łódź_urban	26	0.95	0.95	-0.003	325	330.56	0.404	0.0038
	Łódź_nonurban	24	1.00	0.95	-0.058	276	258.00	0.775	
	Poznań_urban	25	1.00	0.95	-0.050	300	307.78	0.366	
	Poznań_nonurban	25	1.00	0.95	-0.054	300	296.67	0.544	
	Śląsk_urban	24	1.00	0.95	-0.054	276	290.00	0.269	
	Śląsk_nonurban	22	0.95	0.92	-0.033	231	222.50	0.644	
	Warszawa_urban	24	1.00	0.94	-0.062	276	277.11	0.470	
	Warszawa_nonurban	14	0.80	0.88	0.092	91	90.07	0.508	
B106	Łódź_urban	8	0.70	0.77	0.095	28	19.87	0.869	0.1061
	Łódź_nonurban	8	0.90	0.80	-0.127	28	24.02	0.680	
	Poznań_urban	13	0.90	0.86	-0.048	78	65.35	0.846	
	Poznań_nonurban	8	0.80	0.79	-0.019	28	54.72	0.002	
	Śląsk_urban	9	0.80	0.79	-0.014	36	33.38	0.594	
	Śląsk_nonurban	8	0.85	0.79	-0.083	28	21.59	0.800	
	Warszawa_urban	10	0.85	0.82	-0.038	45	33.49	0.897	
	Warszawa_nonurban	8	0.60	0.76	0.214	28	40.64	0.058	
Tm27	Łódź_urban	9	0.85	0.83	-0.029	36	63.81	0.003	-0.0343
	Łódź_nonurban	9	0.85	0.81	-0.054	36	39.95	0.299	
	Poznań_urban	9	0.90	0.83	-0.081	36	26.08	0.888	
	Poznań_nonurban	11	0.80	0.81	0.014	55	55.81	0.444	
	Śląsk_urban	9	0.80	0.83	0.030	36	44.22	0.163	
	Śląsk_nonurban	8	0.85	0.83	-0.021	28	16.37	0.960	
	Warszawa_urban	13	0.85	0.86	0.016	78	48.67	0.996	
	Warszawa_nonurban	7	0.85	0.72	-0.176	21	17.16	0.701	

Table S2. Population differentiation at ADCYAP1, DRD4, CKIε_ex5 and neutral microsatellite markers between eight urban and nonurban populations of the Eurasian coot, as measured with F_{ST} (below diagonal) and Jost's D (above diagonal) values. Significant associations (as inferred based on uncorrected p values) were bolded. Associations which retained statistical significance after FDR correction were indicated with asterisks (*).

		Łódź nonurban	Łódź urban	Poznań nonurban	Poznań urban	Katowice nonurban	Katowice urban	Warszawa nonurban	Warszawa urban
ADCYAP1	Łódź nonurban	-	0.006	0.016	0	0.025	0.010	0	0.032
	Łódź urban	0.004	-	0	0.040	0	0	0	**0.184***
	Poznań nonurban	0.009	0.015	-	0.0002	0.001	0.013	0	**0.185**
	Poznań urban	0.009	0.014	0.002	-	0.022	0.006	0	0.050
	Katowice nonurban	0.014	0.009	0.014	0.016	-	0	0	**0.196***
	Katowice urban	0.012	0.006	0.023	0.019	0.010	-	0	**0.186***
	Warszawa nonurban	0.006	0.004	0.012	0.012	0.004	0.004	-	**0.162***
	Warszawa urban	**0.049**	**0.065**	0.034	0.030	**0.073**	**0.089**	**0.070**	-
DRD4	Łódź nonurban	-	0	0.007	0	0	0.003	0.015	0.017
	Łódź urban	0.008	-	0	0.007	0.008	0.015	0	0
	Poznań nonurban	0.007	0.008	-	0	0.008	0.002	0	0
	Poznań urban	0.008	0.014	0.009	-	0	0	0.009	0.011
	Katowice nonurban	0.015	0.013	0.011	0.006	-	0.007	0.026	0.014
	Katowice urban	0.007	0.013	0.007	0.007	0.010	-	0	0.026
	Warszawa nonurban	0.009	0.018	0.009	0.018	0.023	0.012	-	0.014
	Warszawa urban	0.018	0.016	0.012	0.019	0.021	0.016	0.017	-
CKIε_ex5	Łódź nonurban	-	0	0	0	0	0.0002	0	0.033
	Łódź urban	0.003	-	0	0	0	0.025	0	0.079
	Poznań nonurban	0.002	0.003	-	0	0	0.011	0	0.066
	Poznań urban	0.006	0.002	0.002	-	0	0	0	0.017
	Katowice nonurban	0.004	0.003	0.001	0.001	-	0	0	0.030
	Katowice urban	0.022	0.014	0.012	0.006	0.008	-	0.034	0
	Warszawa nonurban	0.001	0.003	0.003	0.006	0.006	0.024	-	**0.087**
	Warszawa urban	**0.043**	0.026	0.030	0.018	0.023	0.007	**0.040**	-

Neutral microsatellites	Łódź nonurban	-	0.002	0.003	0.003	0.006	**0.065***	0	**0.087***
	Łódź urban	0.013	-	0.019	**0.056***	**0.035***	**0.091***	0.018	**0.095***
	Poznań nonurban	0.013	0.015	-	0.002	**0.026**	**0.031***	0.012	**0.070***
	Poznań urban	0.014	**0.021***	0.013	-	0	**0.068***	0.001	**0.069***
	Katowice nonurban	0.014	**0.019***	**0.017**	0.013	-	0.025	0	**0.081***
	Katowice urban	**0.022***	**0.027***	**0.017***	**0.023***	0.017	-	**0.039***	**0.107***
	Warszawa nonurban	0.012	0.015	0.014	0.013	0.010	**0.018***	-	**0.099***
	Warszawa urban	**0.027***	**0.029***	**0.024***	**0.024***	**0.027***	**0.031***	**0.028***	-

Table S3. Tests for associations between DRD4 genotype and landscape urbanization level in four pairs of urban and nonurban populations of the Eurasian coot. Associations were tested for five most common haplotypes and two single nucleotide polymorphisms (SNP) using additive and overdominant models. Population pair was used as random factor.

	Additive model			Overdominant model		
Polymorphism	Coefficients (mean ± SE)	z	P	Coefficients (mean ± SE)	z	P
DRD4*01	0.64 ± 0.60	1.08	0.281	0.43 ± 0.67	0.65	0.516
DRD4*02	0.08 ± 0.23	0.35	0.726	-0.10 ± 0.32	-0.32	0.751
DRD4*03	0.15 ± 0.54	0.27	0.786	0.15 ± 0.54	0.27	0.786
DRD4*05	-0.97 ± 0.56	-1.75	0.081	-0.97 ± 0.56	-1.75	0.081
DRD4*07	-0.26 ± 0.42	-0.62	0.534	-0.51 ± 0.46	-1.12	0.263
DRD4_SNP321	-0.39 ± 0.36	-1.06	0.290	-0.37 ± 0.43	-0.85	0.393
DRD4_SNP397	-0.32 ± 0.31	-1.05	0.294	-0.07 ± 0.37	-0.18	0.854

Table S4. Tests for associations between ADCYAP1, CKIε_int2 and CKIε_ex5 genotypes and landscape urbanization level in four pairs of urban and nonurban populations of the Eurasian coot. Associations were tested for most common haplotypes and single nucleotide polymorphisms (SNP) using additive and overdominant models. Population pair was used as random factor.

Polymorphism	Additive model			Overdominat model		
	Coefficients (mean ± SE)	z	P	Coefficients (mean ± SE)	z	P
ADCYAP1*145	1.47 ± 0.81	1.82	0.069	-0.26 ± 0.36	-0.72	0.473
ADCYAP1*147	-0.11 ± 0.28	-0.41	0.679	-0.52 ± 0.34	-1.52	0.128
ADCYAP1*149	0.29 ± 0.22	1.31	0.190	-0.10 ± 0.32	-0.32	0.752
ADCYAP1*151	-0.22 ± 0.27	-0.81	0.417	-0.26 ± 0.36	-0.72	0.473
ADCYAP1*153	-0.87 ± 0.56	-1.54	0.123	-0.26 ± 0.36	-0.72	0.473
CKIε_int2*474	0.06 ± 0.25	0.25	0.801	-0.51 ± 0.32	-1.60	0.111
CKIε_int2*476	-0.20 ± 0.26	-0.78	0.438	-0.51 ± 0.32	-1.60	0.111
CKIε_ex5*437	-0.43 ± 0.23	-1.82	0.069	0.00 ± 0.32	0.00	1.000
CKIε_ex5*439	-0.25 ± 0.50	-0.50	0.617	0.25 ± 0.32	0.79	0.429
CKIε_ex5*441	0.40 ± 0.24	1.72	0.086	0.25 ± 0.32	0.79	0.429

Table S5. Results of pairwise post-hoc Wilcoxon comparisons for five regions of behavioral genes and neutral microsatellite markers. Significant associations were bolded.

Gene/Region	pairwise comparisons	χ^2	Df	P
ADCYAP1	**nonurban vs. nonurban**	**8.31**	**1**	**0.004**
	nonurban vs. urban	0.28	1	0.598
	urban vs. urban	0.64	1	0.423
CKIε_int2	**nonurban vs. nonurban**	**8.31**	**1**	**0.004**
	**nonurban vs. urban**	**6.57**	**1**	**0.010**
	**urban vs. urban**	**5.03**	**1**	**0.025**
CKIε_ex5	**nonurban vs. nonurban**	**8.31**	**1**	**0.004**
	**nonurban vs. urban**	**5.46**	**1**	**0.02**
	**urban vs. urban**	**8.31**	**1**	**0.004**
CREB1	**nonurban vs. nonurban**	**5.77**	**1**	**0.016**
	nonurban vs. urban	0.16	1	0.735
	urban vs. urban	2.08	1	0.150
DRD4	nonurban vs. nonurban	0.92	1	0.337
	**nonurban vs. urban**	**21.14**	**1**	**<0.0001**
	**urban vs. urban**	**8.31**	**1**	**0.004**
