# Supplementary material for: Urbanization is associated with non‐coding polymorphisms in candidate behavioural genes in the Eurasian coot
Source: Ecol Evol. 2023 Oct 1;13(10):e10572. doi: 10.1002/ece3.10572 (PMC10542476; doi:10.1002/ece3.10572)

Appendix 2

Urbanization is associated with non-coding polymorphisms in candidate behavioural genes in the Eurasian coot

Amelia Chyb[1], Radosław Włodarczyk[1], Joanna Drzewińska-Chańko[1], Jan Jedlikowski[2], Kimberly K. O. Walden[3], Piotr Minias[1]

1. Department of Biodiversity Studies and Bioeducation, Faculty of Biology and Environmental Protection, University of Łódź, Banacha 1/3, 90-237, Łódź, Poland
2. Faculty of Biology, Biological and Chemical Research Centre, University of Warsaw, Żwirki i Wigury 101, 02-089, Warsaw, Poland
3. Roy J. Carver Biotechnology Center, University of Illinois at Urbana-Champaign, 1206 West Gregory Drive, Urbana, IL 61801, USA.

Correspondence and requests for materials should be addressed to P.M. (email: pminias@op.pl) and A.C. (email: chybamelia@gmail.com)

Figure S1. Frequency of the minor allele of CREB1_SNP216 (A), CREB1_SNP346 (B), and CREB1_SNP380 (C) in four pairs of urban (red) and nonurban (green) populations of the Eurasian coot.

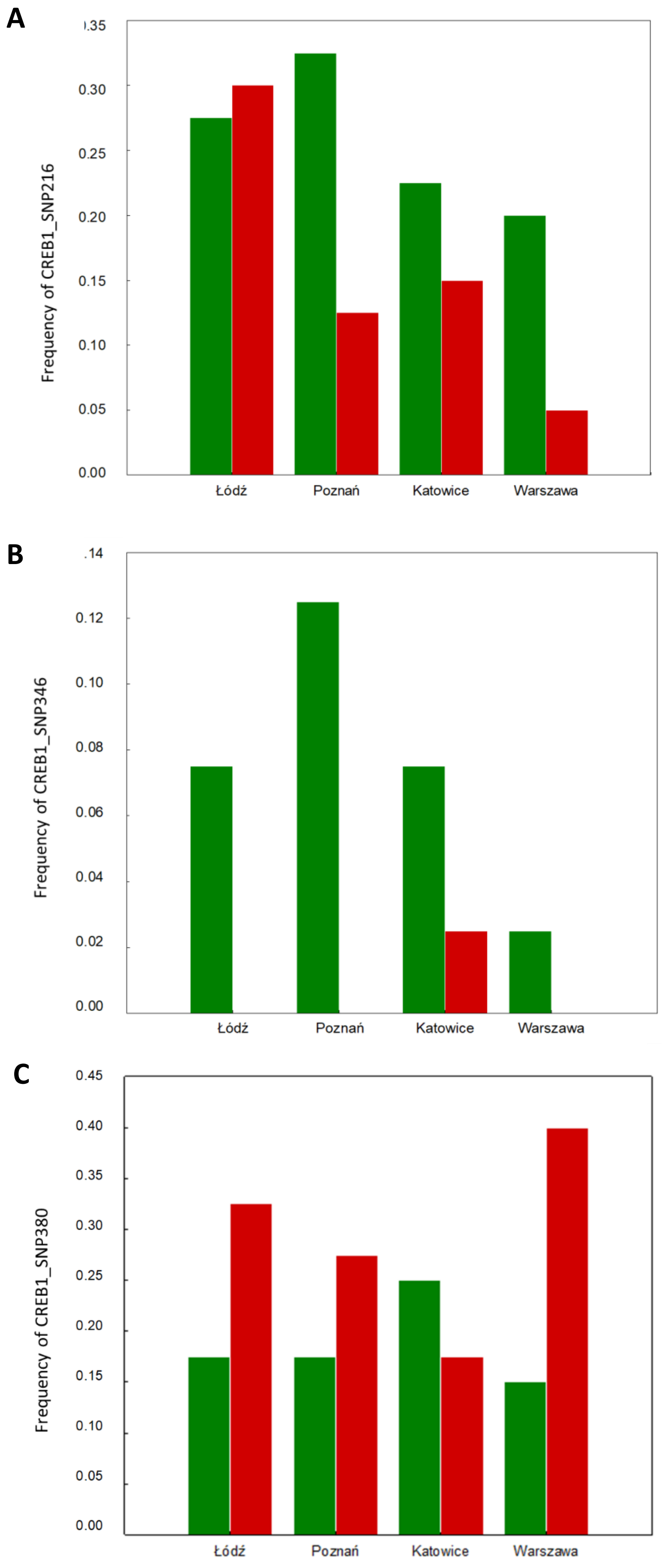

Figure S2. Frequency of CREB1 haplotypes CREB*02 (A) and CREB1*04 (B) in four pairs of urban (red) and nonurban (green) populations of the Eurasian coot.

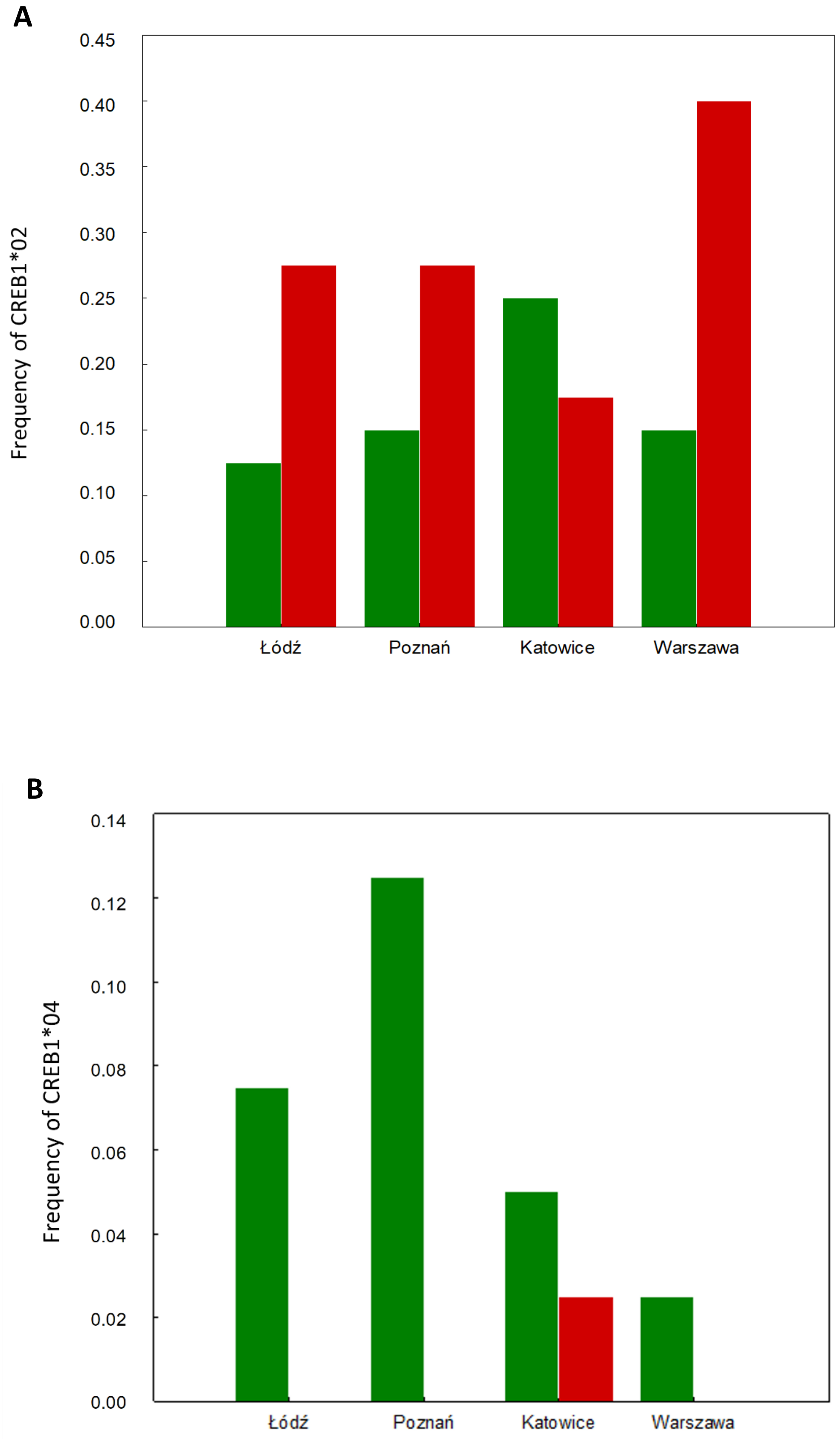

Supplement: Supplementary file 1 — Appendix S1: [file ECE3-13-e10572-s001.zip › ece310572-sup-0002-AppendixS2.pdf]
